# Endemic Burkitt Lymphoma in second-degree relatives in Northern Uganda: in-depth genome-wide analysis suggests clues about genetic susceptibility

**DOI:** 10.1038/s41375-020-01052-w

**Published:** 2020-10-13

**Authors:** Mateus H. Gouveia, Isaac Otim, Martin D. Ogwang, Mingyi Wang, Bin Zhu, Nathan Cole, Wen Luo, Belynda Hicks, Kristine Jones, Kathrin Oehl-Huber, Leona W. Ayers, Stefania Pittaluga, Ismail D. Legason, Hadijah Nabalende, Patrick Kerchan, Tobias Kinyera, Esther Kawira, Glen Brubaker, Arthur G. Levin, Lutz Guertler, Jung Kim, Douglas R. Stewart, Melissa Adde, Ian Magrath, Andrew W. Bergen, Steven J. Reynolds, Meredith Yeager, Kishor Bhatia, Adebowale A. Adeyemo, Ludmila Prokunina-Olsson, Michael Dean, Daniel Shriner, Charles N. Rotimi, Stephen Chanock, Reiner Siebert, Sam M. Mbulaiteye

**Affiliations:** 1 Center for Research on Genomics & Global Health, NHGRI, National Institutes of Health, Bethesda, MD USA; 2 EMBLEM Study, African Field Epidemiology Network, Kampala, Uganda; 3 EMBLEM Study, St. Mary’s Hospital, Lacor, Gulu Uganda; 4 Cancer Genomics Research Laboratory, Leidos Biomedical Research, Frederick National Laboratory for Cancer Research, Frederick, MD USA; 5 Institute of Human Genetics, Ulm University and Ulm University Medical Center, Ulm, Germany; 6 Department of Pathology, The Ohio State University, Columbus, OH USA; 7 Laboratory of Pathology, National Cancer Institute, National Institutes of Health, Bethesda, MD USA; 8 EMBLEM Study, Kuluva Hospital, Arua, Uganda; 9 EMBLEM Study, Shirati Health, Education, and Development Foundation, Shirati, Tanzania; 10 Inter-Church Medical Assistance Mission, Baltimore, MD USA; 11 Max von Pettenkofer Institute, LMU University of München, München, Germany; 12 Division of Cancer Epidemiology and Genetics, National Cancer Institute, National Institutes of Health, US Department of Health and Human Services, Bethesda, MD USA; 13 International Network for Cancer Treatment, Brussels, Belgium; 14 Division of Intramural Research, National Institute of Allergy and Infectious Diseases, National Institutes of Health, US Department of Health and Human Services, Bethesda, MD USA

**Keywords:** Cancer genetics, Cancer genomics

## To the Editor:

Burkitt lymphoma (BL) is an aggressive B-cell lymphoma known to occur as endemic, sporadic, and immunodeficiency types [1]. Endemic Burkitt lymphoma (eBL) has been linked to *Plasmodium falciparum* malaria and Epstein–Barr virus infections [2, 3]. Regardless of type, BL is characterized by hallmark somatic IG-*MYC* chromosomal translocations [1], which cause BL in conjunction with other somatic genetic or epigenetic abnormalities, including in *TP53*, *ID3*, and *TCF3* [4–7]. However, the role of germline genetic susceptibility in BL has not been well studied, although it has been suspected based on reports of familial BL clusters [8].

**Fig. 1:**
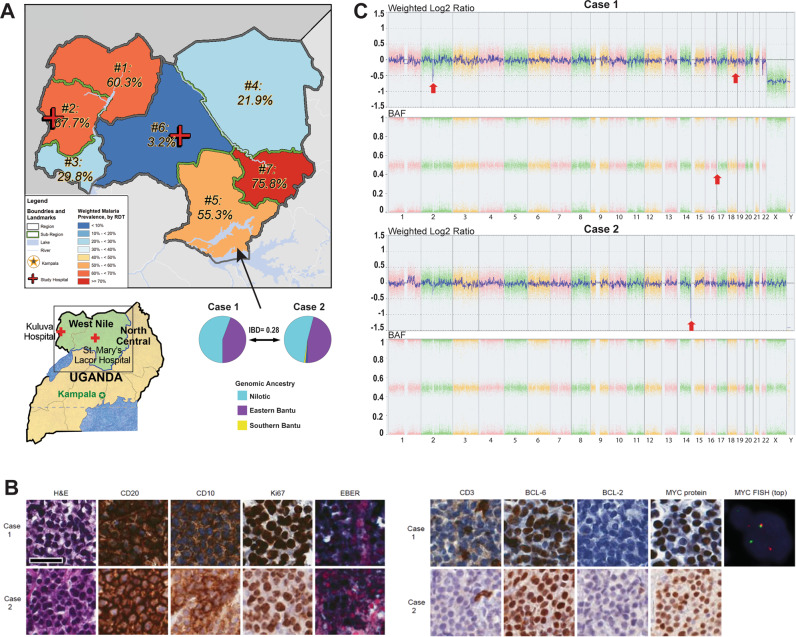
Origin, ancestry, and relatedness of two second-degree relatives with endemic Burkitt lymphoma (eBL) in Northern Uganda. **a** Map of Uganda showing the capital city, Kampala (star with a circle), the boundary of the study area, and the location of the hospitals (red crosses) where the eBL cases were enrolled (see legend). Blue lines in the map mark all-season rivers in Uganda as a surrogate of near-homogenous high precipitation that favors high malaria transmission throughout the country. The zoom-in shows seven subregions (#1–7) in the study area and heterogeneity in prevalence of *Plasmodium falciparum* malaria measured in children aged 0–15 years during the study period (details in Supplementary data [ref. 47]). Pie charts show genomic ancestry and identity by descent (IBD) estimates. The ancestry analysis used the same methodology and African reference populations (details in Gouveia et al. [2]). **b** Pathology and immunohistochemistry (IHC) of eBL tumors. Staining patterns of tumors for Case 1 and Case 2 were similar. The top two rows show: Hematoxylin and Eosin (H&E) atypical lymphocytes, CD20 positive (Dako, Carpinteria, CA, USA), CD10 positive (Novocastra, Bannockburn, IL, USA), Ki67 positive (Dako, Carpinteria, CA, USA), EBER positive (in situ hybridization [ISH]) EBV (Ventana, Tuscon, AZ, USA); The bottom two rows show: CD3 negative in atypical cells (Dako, Carpinteria, CA, USA), BCL-6 positive (Dako, Carpinteria, CA, USA) BCL-2 negative (Dako, Carpinteria, CA, USA), MYC protein positive (Epitomics, Burlingame, CA, USA); MYC translocation positive (only Case 1), fluorescent in situ hybridization probe (FISH) (Vysis LSI MYC Dual Color Break Apart Rearrangement Probe, Abbott Molecular Inc., Des Plaines, IL, USA). Images are ×20 magnification. Scale bar 25 μm. **c** Genome-wide detection of chromosomal imbalances in FFPE tumor tissue by OncoScan Array analysis. For each tumor the imbalance and B-allele frequency plots are shown. The upper panel depicts case 1 (male) in which a loss at the IGK-locus in 2p11 (left arrow), a copy-number neutral loss of 17p13.3p13.1 (including the gene *TP53*, middle arrow) and a heterozygous loss in 18q21.32 (including the gene *CCBE1*, right arrow) were called. The lower panel shows the results of case 2 with loss at the IGH-locus in 14q32 (arrow) and a probable (subclonal) gain in 1q which was just below the diagnostic threshold for calling by the evaluation pipeline.

We report detailed genome-wide analyses in Northern Uganda, including whole-exome sequencing (WES) of eBL tumor and germline DNA (Fig. S1), and genome-wide genotyping array (for Methods see Supplementary data). Based on >4 million variants (Illumina 5 M array) in 198 eBL cases enrolled in the epidemiology of Burkitt lymphoma in East African children and minors (EMBLEM) case–control study (2010–2016) [2, 3], we incidentally discovered two children (one boy and one girl aged 10–15 years) with eBL to be second-degree relatives, likely half-siblings (IBD proportion = 0.28, Fig. 1a).

Using the 198 representative children with confirmed eBL in Northern Uganda as a denominator, we estimate that about 1% of the eBL cases may be genetically related [2]. The two children lived near each other in a high malaria transmission area (Fig. 1a). They presented with a short history of symptoms that started three months apart and were diagnosed with eBL based on histological criteria (Fig. 1b): consistent morphology on hematoxylin and eosin stains, positivity for B cell and germinal center markers, high proliferation index, EBV RNA and MYC translocation. Results of genome-wide copy number variation (CNV) analyses of the tumor DNA based on OncoScan SNP-array (Fig. 1c) and WES (Fig. S2) were consistent with reports that BL has a simple karyotype pattern [5] (Figs. 1c and S2). Specifically, the genomic imbalance mapping of the tumors revealed only a few alterations besides deletions at the IG loci, suggestive of clonal IG rearrangement (Table S1). In the first patient, these changes include apparent copy number neutral loss of heterozygosity (CNN-LOH) in 17p, spanning *TP53*, and a heterozygous loss in 18q21.32, spanning *CCBE1*. Alterations in the second patient were consistent with IG-rearrangement associated loss and a putative low level (sub-clonal) gain in 1q as highly recurrent in BL (Fig. 1c).

This discovery of BL in the two close relatives triggered a review of their medical records, which confirmed Stage C high-risk eBL in both patients. Both responded to treatment (INCTR 03-06 protocol) and were cured (Table 1). Both patients reported their paternal tribe as Langi, which belongs to the Western Nilotic ethno-linguistic group. Consistently, ADMIXTURE analysis showed that both children have more than 50% Nilotic ancestry (Fig. 1a).

**Table 1:** Demographic, clinical, and laboratory characteristics of related Burkitt lymphoma children.

Characteristic	Case 1	Case 2
Demographics		
Season of enrollment	Dry	Wet
Clinical characteristics		
Malaria microscopy/RDT	Negative	Negative
Inpatient malaria treatment	≥12 months ago	Never
Outpatient malaria treatment	≥12 months ago	Never
Fever at enrollment	Yes	Yes
Malaria fever, past 6 months	No	No
Non-malaria fever, past 6 months	Yes	No
Number of fevers, past 12 months	Yes (>6 months ago)	Yes (6 months ago)
Number of admissions	Once	None
Laboratory results (range)		
Hemoglobin, g/dl	10–15	10–15
Total white cell count, cells/10^9^	2.0–2.5	4.0–4.5
Platelet count, cells/10^9^	100–150	300–350
EBV Lei pattern	A	A
Tumor characteristics		
Anatomic sites	Abdomen	Abdomen, jaw
Duration of symptoms	5 months	3 months
Treatment	COM/IT-HR	COM/IT-HR
Number of cycles	6	6
Outcome	Cured	Cured

We analyzed ~400,000 variants in germline WES and 282 mutations identified in tumor WES of the two children (Fig. S1). We focused on candidate germline variants defined as those with moderate-to-high deleterious Combined Annotation Dependent Depletion (CADD) score [9] or those mapped to genes that are recurrently mutated in BL tumors (*n* = 61) or in other cancers (*n* = 180) (Table S2) [5, 6, 10].

We identified 106,404 identical-by-state germline variants (~¼ of the total) in the two children, in agreement with their estimated second-degree of genetic relatedness. Of these variants, 254 were rare variants, with minor allele frequency (MAF) ≤ 0.01 in the ancestrally similar (Nilotic) reference population from Shirati, Tanzania (Tables S3 and S4), and 784 were uncommon variants based on a less stringent MAF ≤ 0.05 (Table S5). Fourteen of the rare variants had a phred-scaled CADD scores >10 (range 10.3–25.3) (Table S3). All the variants were validated by manual review using The Integrative Genomics Viewer (IGV; Fig. S3A). None of the 14 variants were in a region previously suggested to be of regulatory importance in BL based on analysis of differential states of chromatin accessibility in BL-derived cell lines or nonneoplastic B-cells, or showed differential DNA methylation in BL [11] (Table S6). From the 241 candidate genes (Table S2), we identified an intronic SNP (rs772535596) in *CHD8* (Tables S3 and S4), which had a phred-scaled CADD score of ~10, consistent with “moderate evidence of pathogenicity” [12, 13]. *CHD8* was recently identified as recurrently mutated in BL in the BL Genome Sequencing Project [4]. The rs772535596 SNP was not observed in the germline DNA of the unrelated Nilotic individuals from Shirati with BL (*n* = 30) and without BL (*n* = 80).

The discovered 14 candidate variants in 14 genes in the germline DNA of both children (Table S3) were classified as variant of unknown significance (VUS), benign, or likely benign, based on InterVar. Most of the variants were rare (MAF < 1% in the gnomAD database). Of potential importance was an intronic indel rs374301928-ATT/- upstream of exon 12/20 (NM_001369568) in the *TCF4* gene. This variant had a phred-scaled CADD score of 18.7 and “supporting evidence of pathogenicity” according to VarSome [12, 13], although it is currently classified as VUS by InterVar. This variant was also observed in one of the unrelated BL Nilotic patients from Shirati (for a total of three BL cases in our study) but not in any of the unrelated healthy Nilotic individuals from Shirati (*p* = 0.02, Fisherʼs exact test). The rs374301928 variant was observed only nine (0.1%) times in 4,357 African genomes in the gnomAD database (Table S3), suggesting that this is a rare African-specific variant. The variant is found in a highly conserved genomic region, with predicted transcription factor binding sites adjacent to exon 12/20 (Fig. S4). Of interest, the rs374301928-*TCF4* locus appears to have been subject to early negative selection among vertebrate species and archaic hominins that may be indicative of relevant regulatory function [14]. Of pathological relevance to BL, *TCF4* encodes the helix-loop-helix transcription factor 4 reported to interact with *ID3*, which is inactivated by recurrent mutations in up to two-thirds of BL [6, 7, 15]. Somatic *TCF4* deregulation has been implicated as an alternative mechanism of ID3-inactivating or TCF3-activating mutations in BL [6, 15]. Our somatic WES analysis showed that both patients lacked mutations in *ID3* and *TCF3*. Since *TCF4* has been implicated in the *ID3/TCF3* pathway in BL [6, 7, 15], the observed germline/somatic pattern in our cases raises the question of whether germline *TCF4* genetic variants could have an effect comparable to somatic involvement of *ID3/TCF3*. In view of their potential significance, both the *CHD8* and *TCF4* germline variants were verified by Sanger sequencing (Fig. S3B).

The somatic WES analysis for these two children identified 29 mutations in core genes, including *CCND3*, *MYC*, and *USP7* in one child and *BCL7A* and *DDX3X* in the other child, that have been reported to be recurrently mutated in other BL studies (Table S7) [4–7]. Most (266) of the somatic mutations were unique to each child’s tumor (Tables S7 and S8). In addition to the mutations identified in the candidate BL genes, we identified 253 mutations in genes that have not been reported before in BL or other cancers; 70 of these mutations had phred-scaled CADD scores > 20 (Table S8).

While the discovery of genetic relatedness in these two eBL cases suggests a possible genetic predisposition to BL, environmental predisposition from *P. falciparum* malaria and EBV was considered. Both children did not carry common malaria-resistance genetic variants (e.g., the sickle cell trait, see Supplementary data) [3]. One child carried the HLA-B53 allele, previously reported to be associated with resistance to severe malaria in West Africa [16]. Both children were EBV-tumor positive and positive for EBV LMP-1 DNA Pattern A variant (Table 1), which has been associated with a 31-fold higher odds of eBL in EMBLEM [17]. However, the relatively advanced age at BL diagnosis in these children (>10 years) and recent efforts to suppress malaria in their district casts doubt on the hypothesis that these environmental pathogens are the sole triggers.

Our study is a discovery effort with several strengths, including epidemiologically well-characterized samples, extensive genomic data, and availability of tumor tissue. These strengths enabled us to robustly confirm diagnosis by histology and molecular analysis, and to conduct integrated multidisciplinary analysis combining somatic and germline WES data. We confirmed genetic relatedness, ancestry and discovered two variants that warrant follow-up. However, the limitations include small sample size, which precluded consideration of formal statistics (including adjustment for multiple comparisons), and the lack of functional validation of the variants. Also, the paucity of genomic data from individuals in the eBL belt, i.e., Nilotic speakers [2, 3], is a limitation. Our study illustrates the feasibility and scalability of collaborative efforts applying genomic data analysis to identify familial aggregation of eBL in epidemiological or clinical cohorts and that such investigations can shed light on the genetic susceptibility to eBL.

In conclusion, we report the first pathologically confirmed eBL cases in Northern Uganda determined to be related based on their genetic data uncovered and analyzed in the course of an epidemiological study. We identified in both children potentially important germline DNA genetic variants in *TCF4* and *CHD8*. These discoveries, although preliminary, provide novel clues about genetic susceptibility to eBL development.

## Supplementary information

Supplementary material

Si Tables

## Data Availability

The datasets generated and/or analyzed during the current study are available through dbGAP: the EMBLEM data are available through accession: phs001705.v2.p1; the Shirati data are available through accession: phs002223.v1.p1; the Childhood Cancer Survivorship study are available through accession: phs002072.v1.p1; the International Cancer Genome Consortium (ICGC) data were extracted from WGS alignments available from the European Genome-phenome Archive (EGA) under the accession numbers: EGA-S00001002198 in accordance with approval from the ICGC guidelines (www.icgc.org) under DACO-1064755 (National Institutes of Health).

## References

[1] Leoncini L, Campo E, Stein H, Harris NL, Jaffe ES, Kluin PM. Burkitt-like lymphoma with 11q aberration. In: WHO classification of tumours of haematopoietic and lymphoid tissues. Revised 4th ed France, Lyon: IARC; 2017. p. 334.

[2] Gouveia MH, Bergen AW, Borda V, Nunes K, Leal TP, Ogwang MD (2019). Genetic signatures of gene flow and malaria-driven natural selection in sub-Saharan populations of the ‘endemic Burkitt Lymphoma belt’. PLoS Genet.

[3] Legason ID, Pfeiffer RM, Udquim K-I, Bergen AW, Gouveia MH, Kirimunda S (2017). Evaluating the causal link between malaria infection and endemic burkitt lymphoma in Northern Uganda: a mendelian randomization study. EBioMedicine.

[4] Grande BM, Gerhard DS, Jiang A, Griner NB, Abramson JS, Alexander TB (2019). Genome-wide discovery of somatic coding and noncoding mutations in pediatric endemic and sporadic Burkitt lymphoma. Blood.

[5] López C, Kleinheinz K, Aukema SM, Rohde M, Bernhart SH, Hübschmann D (2019). Genomic and transcriptomic changes complement each other in the pathogenesis of sporadic Burkitt lymphoma. Nat Commun.

[6] Richter J, Schlesner M, Hoffmann S, Kreuz M, Leich E, Burkhardt B (2012). Recurrent mutation of the ID3 gene in Burkitt lymphoma identified by integrated genome, exome and transcriptome sequencing. Nat Genet.

[7] Schmitz R, Young RM, Ceribelli M, Jhavar S, Xiao W, Zhang M (2012). Burkitt lymphoma pathogenesis and therapeutic targets from structural and functional genomics. Nature.

[8] Morrow RH, Pike MC, Smith PG, Ziegler JL, Kisuule A (1971). Burkitt’s lymphoma: a time-space cluster of cases in Bwanba County of Uganda. Br Med J.

[9] Rentzsch P, Witten D, Cooper GM, Shendure J, Kircher M (2019). CADD: predicting the deleteriousness of variants throughout the human genome. Nucleic Acids Res.

[10] Waszak SM, Tiao G, Zhu B, Rausch T, Muyas F, Rodriguez-Martin B, et al. Germline determinants of the somatic mutation landscape in 2,642 cancer genomes. bioRxiv. 2017. https://edoc.mdc-berlin.de/17576/. Accessed 14 Jun 2019.

[11] Kretzmer H, Bernhart SH, Wang W, Haake A, Weniger MA, Bergmann AK (2015). DNA methylome analysis in Burkitt and follicular lymphomas identifies differentially methylated regions linked to somatic mutation and transcriptional control. Nat Genet.

[12] Kopanos C, Tsiolkas V, Kouris A, Chapple CE, Albarca Aguilera M, Meyer R (2019). VarSome: the human genomic variant search engine. Bioinformatics.

[13] Richards S, Aziz N, Bale S, Bick D, Das S, Gastier-Foster J (2015). Standards and guidelines for the interpretation of sequence variants: a joint consensus recommendation of the American College of Medical Genetics and Genomics and the Association for Molecular Pathology. Genet Med.

[14] Mozzi A, Forni D, Cagliani R, Pozzoli U, Clerici M, Sironi M (2017). Distinct selective forces and Neanderthal introgression shaped genetic diversity at genes involved in neurodevelopmental disorders. Sci Rep.

[15] Panea RI, Love CL, Shingleton JR, Reddy A, Bailey JA, Moormann AM, et al. The whole genome landscape of Burkitt lymphoma subtypes. Blood. 2019. 10.1182/blood.2019001880. 10.1182/blood.2019001880 PMC6871305 31558468

[16] Hill AV, Elvin J, Willis AC, Aidoo M, Allsopp CE, Gotch FM (1992). Molecular analysis of the association of HLA-B53 and resistance to severe malaria. Nature.

[17] Liao H-M, Liu H, Lei H, Li B, Chin P-J, Tsai S, et al. Frequency of EBV LMP-1 promoter and coding variations in Burkitt lymphoma samples in Africa and South America and peripheral blood in Uganda. Cancers. 2018;10. 10.3390/cancers10060177. 10.3390/cancers10060177 PMC6024959 29865259

