# Supplementary material for: Endemic Burkitt Lymphoma in second-degree relatives in Northern Uganda: in-depth genome-wide analysis suggests clues about genetic susceptibility
Source: Leukemia. 2020 Oct 13;35(4):1209–13. doi: 10.1038/s41375-020-01052-w (PMC8024190; doi:10.1038/s41375-020-01052-w)
Supplement: Supplementary file 1 — Supplementary material [file 41375_2020_1052_MOESM1_ESM.docx]

Supplementary Information

**Endemic Burkitt Lymphoma in second-degree relatives in Northern Uganda:**

**in-depth genome-wide analysis suggests clues about genetic susceptibility**

[Section 1 - Background 2](#_Toc50186000)

[Section 2 - Methods 3](#_Toc50186001)

[*2.1. Ethical Approval of EMBLEM* 3](#_Toc50186002)

[*2.2. Pathology and immunohistochemistry* 3](#_Toc50186003)

[*2.3. DNA extraction and genotyping* 4](#_Toc50186004)

[*2.4. Relatedness and ancestry analyses* 5](#_Toc50186005)

[*2.5. Other samples from non-relatives with or without BL* 5](#_Toc50186006)

[*2.6. Whole Exome Sequencing* 6](#_Toc50186007)

[*2.6.1. DNA Preparation* 6](#_Toc50186008)

[*2.6.2. Pre-Hybridization LM-PCR* 6](#_Toc50186009)

[*2.6.3. Liquid Phase Sequence Capture* 7](#_Toc50186010)

[*2.6.4. Post-Hybridization LM-PCR* 7](#_Toc50186011)

[*2.7. Whole-Exome Sequencing (WES) data and bioinformatic analyses* 8](#_Toc50186012)

[*2.8. Chromosomal imbalance analyses* 10](#_Toc50186013)

[*2.9. Validation by Sanger sequencing* 10](#_Toc50186014)

[Section 3. Supplementary Results 12](#_Toc50186015)

[*3.1. Patient characteristics* 12](#_Toc50186016)

[*3.2. Environmental context of the eBL patients* 13](#_Toc50186017)

[*3.3. Somatic whole-exome sequencing* 14](#_Toc50186018)

[Section 4. Supplementary Discussion 14](#_Toc50186019)

[Section 5. Availability of data and materials 17](#_Toc50186020)

[Section 6. References 17](#_Toc50186021)

[Section 7. Supplementary figures 22](#_Toc50186022)

# Section 1 - Background

Burkitt lymphoma (BL) is an aggressive B-cell lymphoma known to occur in three epidemiological manifestations: sporadic, endemic, and immunodeficiency-related BL [^1^](https://paperpile.com/c/CxF8UM/xOeJ1). Sporadic BL (sBL) is rare (incidence of 1-2 per million) and occurs worldwide; endemic BL (eBL) occurs more commonly in malaria-endemic areas, mostly in Africa (50 per million); and immunosuppression-associated BL (iBL) occurs in the setting of acquired immunodeficiency syndrome (AIDS) or solid organ transplant recipients (at a much higher incidence 600 and 200 per million, respectively) [^2,3^](https://paperpile.com/c/CxF8UM/kfT5P+EH24q). *Plasmodium (P.) falciparum* malaria [^4–6^](https://paperpile.com/c/CxF8UM/x3J2V+U6bdl+6aw8v) and infection with Epstein–Barr virus (EBV) [^7^](https://paperpile.com/c/CxF8UM/wHSQ4) during childhood are established risk factors for BL, particularly in its endemic form. The role of EBV in BL is variable, contributing to 90% of eBL, to 10-30% to sBL and to 40-70% to iBL [^8^](https://paperpile.com/c/CxF8UM/yLT5a). Recent discoveries following integrative genomic and transcriptomic characterization of pediatric eBL and sBL tumors in Africa and North America in the Burkitt Lymphoma Genome Sequencing Project (BLGSP) [^9^](https://paperpile.com/c/CxF8UM/0U2vf) suggest that EBV infection is a marker for BL subgroups with similar molecular patterns in eBL and sBL [^9,10^](https://paperpile.com/c/CxF8UM/0U2vf+tNmAD).

The genetic basis of BL is well-established from hallmark (albeit, not pathognomonic) somatic alterations involving chromosomal translocations that juxtapose the *MYC* oncogene (chromosome 8) with one of three immunoglobulin (*IG*) loci, *IGH*, *IGK,* and *IGL* on chromosomes 14, 2 and 22 respectively, in BL[^11^](https://paperpile.com/c/CxF8UM/NVyLd). These somatic translocations are insufficient to cause BL so they act in conjunction with other somatic genetic or epigenetic abnormalities, including in *MYC*, *TP53*, *ID3*, and *TCF3* [^9,12–14^](https://paperpile.com/c/CxF8UM/0U2vf+Lbj1v+2t5pY+4VIOK), to facilitate progression to BL. Chronic infections with *P. falciparum* malaria and EBV [^7,15,16^](https://paperpile.com/c/CxF8UM/bmirr+wHSQ4+y2lu3) influence BL risk by stimulating dramatic polyclonal expansion of B cells, including those with somatically altered transcriptional programs or epigenetic profiles that favor malignant cellular proliferation [^17^](https://paperpile.com/c/CxF8UM/BV7kz).

However, the role of genetic predisposition from germline variants has not been well studied, although it has been suspected based on reports of epidemiologic clusters of BL among siblings [^18^](https://paperpile.com/c/CxF8UM/ZUVom)^,^[^19^](https://paperpile.com/c/CxF8UM/GieuB). BL in relatives has been reported in malaria-endemic regions, such as the “eBL belt” in Africa [^18,20,21^](https://paperpile.com/c/CxF8UM/AsRMg+ZUVom+j5BUZ) and in Papua New Guinea, and in non-malaria endemic countries such as the United States [^22^](https://paperpile.com/c/CxF8UM/JtOCa) and Denmark [^23^](https://paperpile.com/c/CxF8UM/yucYT). Furthermore, reports of BL complicating rare inherited disorders, including inborn disorders of immune regulation [e.g. Purtillo/Duncan Syndrome/XLP (OMIM: 308240) or XMEN disease (OMIM: 300853)] or DNA repair [e.g. ataxia telangiectasia (OMIM: 615919)], or developmental disorders like Williams-Beuren Syndrome (WBS: OMIM 194050) [^24^](https://paperpile.com/c/CxF8UM/AxNgr), have further underscored the role of genetic predisposition to BL by some germline variants.

# Section 2 - Methods

## *2.1. Ethical Approval of EMBLEM*

The EMBLEM study was approved by the Uganda Virus Research Institute Research and Ethics Committee, the Uganda National Council for Science and Technology, and the NCI Special Studies Institutional Review Boards. Written informed consent was obtained from the guardians of the children, and informed assent was obtained from children aged >8 years old to participate in EMBLEM. No one was re-contacted to discuss relatedness or to collect more contextual information about family relationships and medical history.

## *2.2. Pathology and immunohistochemistry*

Tumor tissues from the two children were reviewed and confirmed as BL by outside pathology at Ohio State University (OSU) for child 1 at OSU and for child 2 at the National Institutes of Health (NIH). Hematoxylin and eosin (H&E) and immunohistochemistry (IHC) tissue staining demonstrated monotonous fields of medium-sized atypical lymphocytes interspersed with macrophages giving the starry–sky pattern observed in rapidly proliferating tumors with apoptosis (Fig 1B). In both children, in situ hybridization (ISH) for detection of Epstein-Barr virus (EBV) EBER RNA was strongly positive and IHC staining for KI67 approached 100%, confirming a high tumor proliferative index in both children. IHC was positive for CD20 staining (B-cell origin) and positive for CD10 and BCL-6 (germinal center cell origin) and negative for CD5 and BCL-2 as expected. CD3 staining revealed a few T cell lymphocytes scattered in the tumors of both children. MYC protein (IHC) was strongly expressed in the atypical lymphocytes in both children [^1^](https://paperpile.com/c/CxF8UM/xOeJ1). The translocation of MYC (FISH) in the atypical cells was demonstrated in patient 1 but this test was not conducted on patient 2 (Fig 1B). Mining of the somatic WES data for potential chimeric or split reads failed to identify in both patients the molecular junctions indicative of a structural variant due to a *MYC*-IG juxtaposition.

## *2.3. DNA extraction and genotyping*

DNA was extracted from previously unthawed buffy coat samples using the Qiagen QIAsymphony automated instrument at the Cancer Genomics Research (CGR) Laboratory (<http://cgf.nci.nih.gov/>), National Cancer Institute (NCI), USA. Genome-wide SNP genotyping was performed at CGR using the Infinium Omni5Exome-4 v1.3 BeadChip (Illumina, San Diego, CA, USA; 4 641 218 SNPs). TaqMan assays for *IL10* rs1800896, *IL1A* rs2856838, and *SEMA3C* rs4461841 or Sanger sequencing (for rs334 in *HBB*) were performed for these previously studied malaria-resistance variants. High-resolution typing of the HLA region (MHC Class I A, B) loci was performed using next-generation sequencing (NGS) technology [^25^](https://paperpile.com/c/CxF8UM/fZknZ).

## *2.4. Relatedness and ancestry analyses*

The relatedness and ancestry analyses were performed in the context of the EMBLEM population genetics study as described in Gouveia et al. 2019 [^5^](https://paperpile.com/c/CxF8UM/U6bdl). Briefly, using a method implemented in PLINK [^26^](https://paperpile.com/c/CxF8UM/eQU2t) and genome-wide data (Illumina Infinium HumanOmni5-4v1), we estimated the identity by descent (IBD), which is the probability at a locus that two alleles, randomly picked from two individuals are inherited from a common ancestor. We performed ancestry analysis using the ADMIXTURE method [^27^](https://paperpile.com/c/CxF8UM/Z0Crz).

## *2.5. Other samples from non-relatives with or without BL*

Given the paucity of sequenced data from cohorts with eBL or from populations from the eBL belt, we generated WES data from ancestry-similar and -dissimilar non-related individuals to facilitate our explorative analysis of germline variants in these two second-degree relatives with eBL. Thus, a total of 242 individuals were sequenced, including 30 non-relatives with eBL and 92 random controls from Shirati in Tanzania. These individuals were sampled in Shirati in Tanzania to study the role of genetic predisposition to eBL [^28^](https://paperpile.com/c/CxF8UM/PrDmB). Shirati is populated by Luo tribes (85% of the population), a Nilotic-speaking group, which migrated from the Sudan regions along the River Nile in Africa, and the Kuria tribe, a mixture of Nilotic- and Bantu-speaking ethno-linguistic groups. In addition, we analyzed WES data from 21 non-relatives, mostly of Mayan descent, with BL from Guatemala [^29^](https://paperpile.com/c/CxF8UM/sC3vB), 65 from the US Childhood Cancer Survivor (CCSS) study [^30^](https://paperpile.com/c/CxF8UM/WqBWe), and 19 from Germany International Cancer Genome Consortium (ICGC) [^12^](https://paperpile.com/c/CxF8UM/Lbj1v). Because clusters of BL cases have been reported in the endemic and non-endemic setting, we also examined germline WES data of BL cases from Guatemala, US, and Germany to determine whether the germline variants identified in the two related eBL cases in Uganda are found in these distinct sporadic settings.

The 92 controls from Shirati were evaluated for genetic relatedness as described in Manichaikul *et al* [^31^](https://paperpile.com/c/CxF8UM/5aojD) implemented in the VCFtools [^32^](https://paperpile.com/c/CxF8UM/sVz8H). Following Kehdy et al. [^33^](https://paperpile.com/c/CxF8UM/9hRNc), we used a kinship coefficient threshold ≥ 0.1 to create family networks, which were analyzed using the optimal algorithm ([maximum clique](https://en.wikipedia.org/wiki/Maximum_clique)) to exclude the minimal number of related individuals that resolve the family structure of the data. Following this procedure, we excluded 12 related individuals and retained 80 unrelated controls as the ancestrally matched (Nilotic) reference population for the analysis of our two eBL related cases.

## *2.6. Whole Exome Sequencing*

### *2.6.1. DNA Preparation*

For each sample, 200 ng of genomic DNA was purified using Agencourt AMPure XP Reagent (Beckman Coulter Life Sciences, Indianapolis, IN) according to manufacturer’s protocol. An adapter-ligated library was prepared with the KAPA HyperPlus Kit (KAPA Biosystems, Wilmington, MA) using xGen Dual Index UMI Adapters (IDT, Coralville, IA) according to KAPA-provided protocol. Fragmentation time was optimized to generate libraries with sequenced insert sizes of 250-350bp.

### *2.6.2. Pre-Hybridization LM-PCR*

Genomic DNA sample libraries were amplified pre-hybridization by ligation-mediated PCR consisting of one reaction containing 20 μL library DNA, 25 μL 2x KAPA HiFi HotStart ReadyMix, and 5μL 10x Library Amplification Primer Mix (primer 1: 5’-AATGATACGGCGACCACCGA-3’ and primer 2: 5’-CAAGCAGAAGACGGCATACGA-3’). PCR cycling conditions were as follows: 98˚C for 45 seconds, followed by 5 cycles of 98˚C for 15 s, 60˚C for 30 s, 72˚C for 30 s, and last extension step at 72˚C for 1 minute. The reaction was kept at 4˚C until further processing. The amplified material was cleaned with Agencourt AMPure XP Reagent (Beckman Coulter Life Sciences, Indianapolis, IN) according to the KAPA-provided protocol. Amplified sample libraries were quantified using Quant-iT™ PicoGreen dsDNA Reagent (ThermoFisher Scientific, Waltham, MA).

### *2.6.3. Liquid Phase Sequence Capture*

Prior to hybridization, amplified sample libraries with unique barcoded adapters were combined in equal amounts into 1.1 μg pools for multiplex sequence capture. Exome sequence capture was performed with Roche NimbleGen’s SeqCap EZ Human Exome Library, either v3.0 with 64 Mb of exonic sequence targeted, or Exome+UTR with 96Mb targeted (Roche NimbleGen, Inc., Madison, WI). Prior to hybridization the following components were added to the 1.1 μg pooled sample library: 2ul xGEN Universal Blocker (IDT), and 5 μL of 1 mg/mL COT-1 DNA (ThermoFisher Scientific). Samples were dried down by puncturing a hole in the plate seal and processing in an Eppendorf 5301 Vacuum Concentrator (Eppendorf, Hauppauge, NY) set to 60˚C for approximately 1 hour. To each dried pool, 7.5 μL of NimbleGen Hybridization Buffer and 3.0 μL of NimbleGen Hybridization Component A were added, and placed in a heating block for 10 minutes at 95˚C. The mixture was then transferred to 4.5 μL of EZ Exome Probe Library and hybridized at 47˚C for 64 to 72 hours. Washing and recovery of captured DNA were performed as described in NimbleGen SeqCap EZ Library SR Protocol.

### *2.6.4. Post-Hybridization LM-PCR*

Pools of captured DNA were amplified by ligation-mediated PCR consisting of one reaction for each pool containing 20μl captured library DNA, 25 μL 2x KAPA HiFi HotStart ReadyMix, and 5μL 10x Library Amplification Primer Mix primer 1: 5’-AATGATACGGCGACCACCGA-3’ and primer 2: 5’-CAAGCAGAAGACGGCATACGA-3’). PCR cycling conditions were as follows: 98˚C for 45 seconds, followed by 8 cycles of 98˚C for 15 s, 60˚C for 30 s, 72˚C for 30 s, with last extension step at 72˚C for 1 minute. The reaction was kept at 4˚C until further processing. The amplified material was cleaned with Agencourt AMPure XP Reagent according to NimbleGen SeqCap EZ Library SR Protocol. Pools of amplified captured DNA were then quantified via Kapa’s Library Quantification Kit for Illumina (Kapa Biosystems) on the LightCycler 480 (Roche, Indianapolis, IN).

## *2.7. Whole-Exome Sequencing (WES) data and bioinformatic analyses*

WES was performed at the CGR, NCI with SeqCap EZ Human Exome v3.0 (Roche/Nimblegen) as described elsewhere [^34,35^](https://paperpile.com/c/CxF8UM/VLe3B+21aa4). Pools of captured DNA underwent paired-end sequencing using an Illumina HiSeq according to Illumina-provided protocols for 2 × 150-bp paired-end sequencing. Each exome was sequenced to high depth to achieve a minimum threshold of 80% of the coding sequence covered by at least 15 reads, based on the UCSC hg19 ‘known gene’ transcripts. Sequencing quality control, alignment, variant discovery, and genotype calling were done as previously described [^36^](https://paperpile.com/c/CxF8UM/t9x0g). Briefly, we used the reference genome version hg19 to perform initial quality control, followed by alignment and variant discovery. Our pipelines of analyses of germline and somatic data are described in Fig. S1A and B, respectively.

To improve variant calling, we combined all the data for the eBL children with germline WES or whole-genome sequencing (reduced to exome content) for 135 other BL patients from different geographical regions (US, Germany, Guatemala, and Tanzania) and 80 unrelated Nilotic healthy controls from Tanzania. We used HaplotypeCaller (GATK v3.8-1) [^37^](https://paperpile.com/c/CxF8UM/M5HNZ) with default parameters to call the ES germline data. To remove possible variant artifacts, we applied post-calling GATK “Hard Filtering” separately for SNPs and indels [^37^](https://paperpile.com/c/CxF8UM/M5HNZ). Furthermore, we used bcftools [^38^](https://paperpile.com/c/CxF8UM/yT7fm) to exclude variants with an alternative allele depth less than ten. Next, we filtered the common variants (i.e. identical in state) between the eBL children. From these variants, we retained only those with a minor allele frequency of < 1% in our reference control population from Shirati. We performed functional annotation of the following components: i) basic annotation (gene, function and functional impact); ii) [Combined Annotation Dependent Depletion](https://cadd.gs.washington.edu/) (CADD) scores; iii) methylation and chromatin states using epigenomic data from non-neoplastic lymphocytes and BL cells [^39^](https://paperpile.com/c/CxF8UM/pVAvd); and iv) gene expression and v) e[xpression quantitative trait loci](https://en.wikipedia.org/wiki/Expression_quantitative_trait_loci) (eQTL) identification using GTEx V7 data (<https://gtexportal.org/home/datasets>). Then, we split the variants into three groups: i) variants within genes previously reported to be somatically mutated in BL [^12–14^](https://paperpile.com/c/CxF8UM/2t5pY+4VIOK+Lbj1v), ii) variants within genes previously reported to be somatically mutated in cancer [^40^](https://paperpile.com/c/CxF8UM/gWKxo), and iii) variants with a depletion score (CADD) > 10, which was considered sufficient for our exploratory purposes. Together these genes constituted our BL candidate genes. Finally, all the remaining variant calls were visualized using The Integrative Genomics Viewer (IGV) [^41^](https://paperpile.com/c/CxF8UM/iPknT) and those considered artifacts excluded.

Somatic variant calling (Fig. S1B) was performed using TNsnv (matches GATK’s Mutect) and TNhaplotyper (matches GATK’s Mutect2) [^42^](https://paperpile.com/c/CxF8UM/hV9w1) from the commercial software Sentieon (Release 201711.03). For both callers, we used the default parameters and supplied COSMIC v86 and dbSNP build 138 and the Pan-Cancer Analysis of Whole Genomes (PCAWG) germline variation callset [^40^](https://paperpile.com/c/CxF8UM/gWKxo) as the Panel of Normals (PONs). Calling was performed using the normal-tumor strategy, in which the normal (germline) data were used as a reference to call only the somatic mutations not observed in the germline data but observed in the tumor (somatic) data. To remove possible false positives, we only considered concordant mutations called by both somatic callers. Then, we performed functional annotation as described above for the germline pipeline of analysis and split in the variants into three groups: i) those in putative BL genes, ii) those in putative cancer genes and iii) those with high CADD scores. Variant were classified into five categories, P (pathogenic), LP (likely pathogenic), VUS (variant of unknown significance), LB (likely benign), and B (benign), using a hierarchical system: ClinVar [^43^](https://paperpile.com/c/CxF8UM/Q320M) classification based on clinical laboratories meeting minimum requirements for data sharing to support quality assurance by ClinGen (<https://www.clinicalgenome.org/lablist>) followed by InterVar version 2.1.2 [^44^](https://paperpile.com/c/CxF8UM/vQYnl).

## *2.8. Chromosomal imbalance analyses*

FFPE derived tumor DNA from both patients was hybridized to an OncoScan CNV assay (Thermo Fisher Scientific, Waltham, MA, USA) to detect genome-wide copy number and loss-of-heterozygosity alterations. Analysis was performed using the Chromosome Analysis Suite Software version 4.0 (Thermo Fisher Scientific, Waltham, MA, USA). Only copy number alterations larger than 50 kb, encompassing at least 20 informative probes with a median log_2_ratio of >0.3 or <-0.3 were considered for further analysis. Moreover, only copy number neutral losses of heterozygosity larger than 5 Mb were considered. All variants were manually inspected and curated considering the B-allele-frequency and the sample quality. We also performed an allele-specific copy number analysis of the somatic WES data using FACETS [^45^](https://paperpile.com/c/CxF8UM/0tRyF) (Fraction and Allele-Specific Copy Number Estimates from Tumor Sequencing).

## *2.9. Validation by Sanger sequencing*

Sanger sequencing was performed at the CGR using the BigDye® Terminator v3.1 Cycle Sequencing reagents (ThermoFisher, Waltham, MA). Sanger sequencing, which provides a high degree of accuracy for determination of SNPs and indels from germline DNA, found no discrepancies with WES.

The resulting post-capture enriched multiplexed sequencing libraries were loaded on a NovaSeq 6000 (Illumina, San Diego, CA) and paired-end sequencing was performed using read lengths of 2x150bp.

The regions of interest were PCR-amplified with the following primers from IDT, Coralville, IA:

*TCF4*, rs374301928, chr 18, 270 bp amplicon, Forw: cccgcttcctctatttgctg; Rev: cagcaaggggaaagtcatagc

*CHD8*, rs772535596, chr 14, 464 bp amplicon, Forw: cagtgtccagctcctccata; Rev: gagctgtgatagggccatga

PCR reactions included: 4ul of 5ng/ul genomic DNA, 5ul AmpliTaq Gold 360 Master Mix (Thermofisher), and 1ul of 5uM primer mix (forward and reverse primers) in 10 ul and were performed at 95˚C hold for 10 min, 40 cycles of 95˚C for 30 sec, 60˚C for 45 sec, and 72 ˚C for 45 sec, with a final 72 ˚C hold of 3 min. The PCR products were purified with Agencourt AMPure XP Reagent (Beckman Coulter, Brea, CA) and sequenced separately with the forward and reverse PCR primers, using 1ul of purified PCR products and 1ul BigDye Terminator v3.1 Ready Reaction Mix (ThermoFisher). The reactions were purified using Agencourt CleanSeq (Beckman Coulter), sequenced on the 3730xl DNA Analyzer, and analyzed using Sequencher 4.8 (Gene Codes, Ann Arbor, MI).

# Section 3. Supplementary Results

## *3.1. Patient characteristics*

Using genome-wide single nucleotide polymorphism (SNP) data, we identified two second-degree relatives (IBD=0.28) from among 198 children with confirmed eBL (i.e. ~1% of cases) in Northern Uganda [^5^](https://paperpile.com/c/CxF8UM/U6bdl). The two children lived near each other in North-Central Uganda and were diagnosed with eBL three months apart (Fig 1A; Table 1) [^46^](https://paperpile.com/c/CxF8UM/7SHjv). The local eBL diagnosis was confirmed by outside pathology review (Fig 1B).

***Patient 1:*** A child aged 10-15 years was diagnosed between 2010-2016 with Ziegler Stage C high-risk (HR) eBL following a five-month history of abdominal swelling, pain, and fatigue. No family history of cancer was reported. Laboratory tests showed serum lactate dehydrogenase (LDH) was >1000 IU/L (reference range 225-450 U/L), cerebrospinal fluid (CSF) was normal, hemoglobin was >10 g/dL, and antibody tests for hepatitis B surface antigen and HIV were negative. The patient received six chemotherapy cycles according to the INCTR 03-06 protocol, achieved remission, and is alive and well seven years later.

***Patient 2:*** A child aged 10-15 years was diagnosed between 2010-2016 with Ziegler Stage C HR eBL following a three-month history of progressive abdominal and left jaw swelling and fever. Laboratory tests showed serum LDH was >2000 U/L, CSF was normal, hemoglobin was >10 g/dL, and antibody tests for hepatitis B surface antigen and HIV were negative. Bone marrow examination was not done. The patient received six chemotherapy cycles according to the INCTR 03-06 protocol, achieved remission, and is alive and well seven years later.

These two children were identified to be genetically related based on genome-wide SNP data analysis of 198 eBL cases from northern Uganda [^5,6^](https://paperpile.com/c/CxF8UM/U6bdl+6aw8v). The children were identified as second-degree relatives, likely half-siblings, based on genomic analysis of identity by descent (IBD), with the proportion of the genome shared IBD estimated to be 0.28 and Pr(IBD=0) = 0.45, Pr(IBD=1) = 0.55, and Pr(IBD=2) = 0. This discovery triggered a review of their records, which confirmed that their relatedness was not reported during enrollment. Privacy and confidentiality concerns precluded any consideration of contacting the families to clarify their social relatedness. Chromosome X analysis showed that the children did not share their X chromosomes. This excludes an X-linked inheritance pattern (including e.g. XLP). Moreover, the children did not exhibit dysmorphic features for X-linked inherited syndromes.

## *3.2. Environmental context of the eBL patients*

Both children lived in a high malaria transmission area [^47^](https://paperpile.com/c/CxF8UM/jf1qT). Although targeted for malaria suppression using indoor residual insecticide spraying (IRS) during 2009-2012, the *P. falciparum* malaria prevalence (*pf*PR) in children aged 0-15 years in this area was several-fold higher (55.3% versus 3.2-19%; Fig. 1A) than that in neighboring regions where malaria was also suppressed [^47^](https://paperpile.com/c/CxF8UM/jf1qT). Despite the high malaria prevalence in their area, both children reported no or minimal malaria episodes in the 12 months before eBL onset and were negative for malaria at enrollment [^6^](https://paperpile.com/c/CxF8UM/6aw8v). The children did not carry common malaria-resistance genetic variants [rs334-*HBB* (T>A), rs1800896- *IL10* (T>C)], which is consistent with the hypothesis that non-carriage of malaria resistance genetic variants is a risk factor for eBL [^6^](https://paperpile.com/c/CxF8UM/6aw8v). One child carried the HLA-B53 allele, which has been associated with resistance to severe malaria in West Africa [^48^](https://paperpile.com/c/CxF8UM/1TTmN) but not in East Africa. Both children were positive for EBV LMP-1 DNA Pattern A variant (Table 1), which has been associated with a 31-fold higher odds ratio of eBL in EMBLEM [^49^](https://paperpile.com/c/CxF8UM/riU1J).

## *3.3. Somatic whole-exome sequencing*

Tumors in both children lacked mutations in *ID3* and *TCF3*. Four BL candidate genes (*ACTB, CSMD1, DNAH10,* and *BMP7*) were somatically involved in both children but with unique mutations (Table S7).

Three BL candidate genes (*CCND3, KMT2D*, and *USP7*) carried somatic stop-gain somatic mutations with high deleteriousness scores (phred-scaled CADD> 37). Although mutations in *CCND3* are rare (1.8%) in EBV-positive or eBL patients [^9^](https://paperpile.com/c/CxF8UM/0U2vf), they were present in one of the eBL children. Mutations in *USP7* were recently identified to be recurrently mutated in BL in the BLGSP [^9^](https://paperpile.com/c/CxF8UM/0U2vf); USP7 is a de-ubiquitinating enzyme that regulates the p53-MDM2 pathway. The Epstein-Barr virus nuclear antigen 1 protein binds with high affinity to USP7 and disrupts the USP7-p53 interaction, potentially contributing to EBV-mediated lymphomagenesis [^56^](https://paperpile.com/c/CxF8UM/RAN6r).

# Section 4. Supplementary Discussion

We conducted an in-depth genetic analysis of two second-degree relatives with histologically confirmed eBL from Northern Uganda [^5,6^](https://paperpile.com/c/CxF8UM/6aw8v+U6bdl) to obtain clues about genetic predisposition to eBL. Based on two related cases identified in a set of 198 eBL patients, we estimate that eBL in genetically related individuals could contribute ~ 1% of eBL cases in Northern Uganda. This figure is likely an underestimate because cases that die early are likely to be missed. Our data are also incomplete regarding social context and generation relationships (half-siblings or double first cousins who come from the same generation or grandparent-grandchild or aunt/uncle-niece/nephew relationships, who come from a different generation).

The novel findings in our study are the germline DNA variants in *TCF4* and *CHD8*, which are plausible BL candidate susceptibility genes. We considered the intronic deletion (rs374301928) in *TCF4* to be a strong clue because: i) the locus is conserved over many species, including early modern humans [^55^](https://paperpile.com/c/CxF8UM/69CZT), suggesting that it has been selected for biological function; ii) it is associated with a moderate-to-high phred-scaled CADD score [^50^](https://paperpile.com/c/CxF8UM/fjAkA) (Table S3); iii) despite the rarity of the variant (only nine deletions observed in 4,357 African genomes [^53^](https://paperpile.com/c/CxF8UM/ExVXj)), it was found in the germline DNA of the two related eBL cases and in one eBL case from Shirati, but not in 80 ancestrally matched (Nilotic) healthy controls. Furthermore, the tumors of both related eBL cases carrying the rs374301928 variant in the germline DNA lacked any detectable somatic mutations in *TCF3* and *ID3*, frequently altered in sBL tumors and to a lesser extent in eBL [^12–14^](https://paperpile.com/c/CxF8UM/Lbj1v+2t5pY+4VIOK). Since *TCF4* has been implicated in the *ID3/TCF3* pathway involved in BL [^10,13,14^](https://paperpile.com/c/CxF8UM/tNmAD+2t5pY+4VIOK), the observed pattern raises the question whether germline *TCF4* genetic variants could have an effect comparable to somatic involvement of *ID3/TCF3*. We evaluated the variant rs772535596 in *CHD8* as a suggestive finding because it is recurrently mutated in BL [^9^](https://paperpile.com/c/CxF8UM/0U2vf). Furthermore, *CHD8* encodes for a protein that is a negative regulator of the WNT-β-catenin signaling pathway [^57^](https://paperpile.com/c/CxF8UM/loot7) and is hypothesized to disrupt epigenetic regulation [^9^](https://paperpile.com/c/CxF8UM/0U2vf).

Although the diagnosis of eBL in two related children could be due to chance, we considered the geographical proximity and temporal proximity (just three months apart) to be epidemiological clues about genetic susceptibility acting in conjunction with an environmental trigger. Such a scenario (environmental trigger in susceptible individuals) was proposed to explain BL clusters reported in siblings in Uganda in the 1960s [^18^](https://paperpile.com/c/CxF8UM/ZUVom) and in the US in the 1970s [^58^](https://paperpile.com/c/CxF8UM/cjVU1). Plausibly, *P. falciparum* malaria and EBV could be the environmental triggers in the two children we report. Both were susceptible to malaria because they lacked genetic variants that protect against malaria (e.g., the sickle cell trait) and both children were positive for the EBV LMP-1 Pattern A variant, which has been associated with a 31-fold higher risk for eBL [^49^](https://paperpile.com/c/CxF8UM/riU1J). However, given that children are typically first exposed to *P. falciparum* malaria and EBV before their first birthday, we doubt that such early exposure to *P. falciparum* malaria or EBV is the trigger of eBL ten to 12 years later in these children. We make a possible exception for a role of rare (presumably, high-risk) strains of *P. falciparum* malaria or EBV infections, which might be encountered later in childhood because of their low frequency and could triggers eBL in susceptible children [^59^](https://paperpile.com/c/CxF8UM/e6Z7a).

Our study has several strengths. The most important strength was having epidemiologically well-characterized samples and genome-wide SNP data which enabled us to conduct an integrated multi-disciplinary analysis to document relatedness, ancestry, and identify clues about susceptibility. Further, availability of tumor tissue enabled us to confirm the diagnosis by outside histological and molecular analysis. Our study also has limitations. The small sample size, lack of samples from non-affected family members, and the paucity of genomic data from individuals in the eBL belt, i.e., Nilotic speakers [^5,6^](https://paperpile.com/c/CxF8UM/U6bdl+6aw8v) , are limitations.

In conclusion, we report the identification of second-degree relatives with confirmed eBL in Northern Uganda. We discovered in germline DNA of both children genetic variants in *TCF4* and *CHD8*, which could be involved in susceptibility to eBL development, perhaps in combination with environmental factors (*P. falciparum* malaria, EBV, or an unidentified pathogen). These preliminary results provide a first step towards understanding susceptibility to eBL, which should be followed up in larger sample size studies or studies using other methods such as genome-wide association studies as is currently planned in the EMBLEM project.

# Section 5. Availability of data and materials

The datasets generated and/or analyzed during the current study are available through dbGAP (The EMBLEM data are available through accession: phs001705.v2.p1; the Shirati data are available through accession: phs002223.v1.p1; the Childhood Cancer Survivorship study are available through accession: phs002072.v1.p1; The International Cancer Genome Consortium (ICGC) data were extracted from WGS alignments are available from the European Genome-phenome archive (EGA) under the accession numbers: EGA-S00001002198 in accordance with approval from the ICGC guidelines ([www.icgc.org](http://www.icgc.org)) under DACO-1064755 (National Institutes of Health).

# Section 6. References

1 [Leoncini L, Campo E, Stein H, Harris NL, Jaffe ES, Kluin PM. Burkitt-like lymphoma with 11q aberration. *WHO classification of tumours of haematopoietic and lymphoid tissues Revised 4th ed France, Lyon: IARC* 2017; : 334.](http://paperpile.com/b/CxF8UM/xOeJ1)

2 [Mbulaiteye SM, Clarke CA, Morton LM, Gibson TM, Pawlish K, Weisenburger DD *et al.* Burkitt lymphoma risk in U.S. solid organ transplant recipients. *Am J Hematol* 2013; **88**: 245–250.](http://paperpile.com/b/CxF8UM/kfT5P)

3 [Guech-Ongey M, Simard EP, Anderson WF, Engels EA, Bhatia K, Devesa SS *et al.* AIDS-related Burkitt lymphoma in the United States: what do age and CD4 lymphocyte patterns tell us about etiology and/or biology? *Blood* 2010; **116**](http://paperpile.com/b/CxF8UM/EH24q): 5600–5604.

4 [Aka P, Vila MC, Jariwala A, Nkrumah F, Emmanuel B, Yagi M *et al.* Endemic Burkitt lymphoma is associated with strength and diversity of Plasmodium falciparum malaria stage-specific antigen antibody response. *Blood* 2013; **122**: 629–635.](http://paperpile.com/b/CxF8UM/x3J2V)

5 [Gouveia MH, Bergen AW, Borda V, Nunes K, Leal TP, Ogwang MD *et al.* Genetic signatures of gene flow and malaria-driven natural selection in sub-Saharan populations of the ‘endemic Burkitt Lymphoma belt’. *PLoS Genet* 2019; **15**: e1008027.](http://paperpile.com/b/CxF8UM/U6bdl)

6 [Legason ID, Pfeiffer RM, Udquim K-I, Bergen AW, Gouveia MH, Kirimunda S *et al.* Evaluating the Causal Link Between Malaria Infection and Endemic Burkitt Lymphoma in Northern Uganda: A Mendelian Randomization Study. EBioMedicine. 2017; **25**: 58–65.](http://paperpile.com/b/CxF8UM/6aw8v)

7 [de-Thé G, Geser A, Day NE, Tukei PM, Williams EH, Beri DP *et al.* Epidemiological evidence for causal relationship between Epstein-Barr virus and Burkitt’s lymphoma from Ugandan prospective study. Nature. 1978; **274**: 756–761.](http://paperpile.com/b/CxF8UM/wHSQ4)

8 [Mbulaiteye SM, Pullarkat ST, Nathwani BN, Weiss LM, Rao N, Emmanuel B *et al.* Epstein-Barr virus patterns in US Burkitt lymphoma tumors from the SEER residual tissue repository during 1979-2009. APMIS. 2014; **122**: 5–15.](http://paperpile.com/b/CxF8UM/yLT5a)

9 [Grande BM, Gerhard DS, Jiang A, Griner NB, Abramson JS, Alexander TB *et al.* Genome-wide discovery of somatic coding and noncoding mutations in pediatric endemic and sporadic Burkitt lymphoma. *Blood* 2019; **133**: 1313–1324.](http://paperpile.com/b/CxF8UM/0U2vf)

10 [Panea RI, Love CL, Shingleton JR, Reddy A, Bailey JA, Moormann AM *et al.* The whole genome landscape of Burkitt lymphoma subtypes. *Blood* 2019. doi:](http://paperpile.com/b/CxF8UM/tNmAD)[10.1182/blood.2019001880](http://dx.doi.org/10.1182/blood.2019001880)[.](http://paperpile.com/b/CxF8UM/tNmAD)

11 [Dalla-Favera R, Bregni M, Erikson J, Patterson D, Gallo RC, Croce CM. Human c-myc onc gene is located on the region of chromosome 8 that is translocated in Burkitt lymphoma cells. *Proc Natl Acad Sci U S A* 1982; **79**: 7824–7827.](http://paperpile.com/b/CxF8UM/NVyLd)

12 [López C, Kleinheinz K, Aukema SM, Rohde M, Bernhart SH, Hübschmann D *et al.* Genomic and transcriptomic changes complement each other in the pathogenesis of sporadic Burkitt lymphoma. *Nat Commun* 2019; **10**: 1459.](http://paperpile.com/b/CxF8UM/Lbj1v)

13 [Richter J, Schlesner M, Hoffmann S, Kreuz M, Leich E, Burkhardt B *et al.* Recurrent mutation of the ID3 gene in Burkitt lymphoma identified by integrated genome, exome and transcriptome sequencing. *Nat Genet* 2012; **44**: 1316–1320.](http://paperpile.com/b/CxF8UM/2t5pY)

14 [Schmitz R, Young RM, Ceribelli M, Jhavar S, Xiao W, Zhang M *et al.* Burkitt lymphoma pathogenesis and therapeutic targets from structural and functional genomics. *Nature* 2012; **490**: 116–120.](http://paperpile.com/b/CxF8UM/4VIOK)

15 [Chakravorty S, Yan B, Wang C, Wang L, Quaid JT, Lin CF *et al.* Integrated Pan-Cancer Map of EBV-Associated Neoplasms Reveals Functional Host-Virus Interactions. *Cancer Res* 2019. doi:](http://paperpile.com/b/CxF8UM/bmirr)[10.1158/0008-5472.CAN-19-0615](http://dx.doi.org/10.1158/0008-5472.CAN-19-0615)[.](http://paperpile.com/b/CxF8UM/bmirr)

16 [Hernandez-Vargas H, Gruffat H, Cros MP, Diederichs A, Sirand C, Vargas-Ayala RC *et al.* Viral driven epigenetic events alter the expression of cancer-related genes in Epstein-Barr-virus naturally infected Burkitt lymphoma cell lines. *Sci Rep* 2017; **7**: 5852.](http://paperpile.com/b/CxF8UM/y2lu3)

17 [Kreck B, Richter J, Ammerpohl O, Barann M, Esser D, Petersen BS *et al.* Base-pair resolution DNA methylome of the EBV-positive Endemic Burkitt lymphoma cell line DAUDI determined by SOLiD bisulfite-sequencing. *Leukemia* 2013; **27**: 1751–1753.](http://paperpile.com/b/CxF8UM/BV7kz)

18 [Morrow RH, Pike MC, Smith PG, Ziegler JL, Kisuule A. Burkitt’s Lymphoma: A Time-space Cluster of Cases in Bwanba County of Uganda. *Br Med J* 1971; **2**: 491–492.](http://paperpile.com/b/CxF8UM/ZUVom)

19 [Li FP. Familial Burkitt lymphoma and nasopharngeal carcinoma. *Lancet* 1976; **1**: 687–688.](http://paperpile.com/b/CxF8UM/GieuB)

20 [Salawu L, Fatusi OA, Kemi-Rotimi F, Adeodu OO, Durosinmi MA. Familial Burkitt’s lymphoma in Nigerians. *Ann Trop Paediatr* 1997; **17**: 375–379.](http://paperpile.com/b/CxF8UM/AsRMg)

21 [Williams EH, Smith PG, Day NE, Geser A, Ellice J, Tukei P. Space-time clustering of Burkitt’s lymphoma in the West Nile district of Uganda: 1961-1975. *Br J Cancer* 1978; **37**: 109–122.](http://paperpile.com/b/CxF8UM/j5BUZ)

22 [Anderson KC, Jamison DS, Peters WP, Li FP. Familial Burkitt’s lymphoma. Association with altered lymphocyte subsets in family members. *Am J Med* 1986; **81**: 158–162.](http://paperpile.com/b/CxF8UM/JtOCa)

23 [Poulsen LO, Christensen JH, Sørensen B, Ebbesen P, Pallesen G, Grunnet N. Immunologic observations in close relatives of two sisters with mammary Burkitt’s lymphoma. Mammary Burkitt's lymphoma in sisters. *Cancer* 1991; **68**: 1031–1034.](http://paperpile.com/b/CxF8UM/yucYT)

24 [Kimura R, Ishii Y, Tomiwa K, Awaya T, Nakata M, Kato T *et al.* Williams-Beuren Syndrome as a Potential Risk Factor for Burkitt Lymphoma. *Front Genet* 2018; **9**: 368.](http://paperpile.com/b/CxF8UM/AxNgr)

25 [Kirimunda S, Verboom M, Otim I, Ssennono M, Legason ID, Nabalende H *et al.* Variation in the Human Leukocyte Antigen system and risk for endemic Burkitt lymphoma in northern Uganda. *Br J Haematol* 2020; **189**: 489–499.](http://paperpile.com/b/CxF8UM/fZknZ)

26 [Purcell S, Neale B, Todd-Brown K, Thomas L, Ferreira MAR, Bender D *et al.* PLINK: a tool set for whole-genome association and population-based linkage analyses. *Am J Hum Genet* 2007; **81**: 559–575.](http://paperpile.com/b/CxF8UM/eQU2t)

27 [Alexander DH, Novembre J, Lange K. Fast model-based estimation of ancestry in unrelated individuals. *Genome Res* 2009; **19**: 1655–1664.](http://paperpile.com/b/CxF8UM/Z0Crz)

28 [Brubaker G, Levin AG, Steel CM, Creasey G, Cameron HM, Linsell CA *et al.* Multiple cases of Burkitt’s lymphoma and other neoplasms in families in the North Mara District of Tanzania. *Int J Cancer* 1980; **26**: 165–170.](http://paperpile.com/b/CxF8UM/PrDmB)

29 [Dean M, Bendfeldt G, Lou H, Giron V, Garrido C, Valverde P *et al.* Increased incidence and disparity of diagnosis of retinoblastoma patients in Guatemala. *Cancer Lett* 2014; **351**: 59–63.](http://paperpile.com/b/CxF8UM/sC3vB)

30 [Morton LM, Sampson JN, Armstrong GT, Chen T-H, Hudson MM, Karlins E *et al.* Genome-Wide Association Study to Identify Susceptibility Loci That Modify Radiation-Related Risk for Breast Cancer After Childhood Cancer. *J Natl Cancer Inst* 2017; **109**. doi:](http://paperpile.com/b/CxF8UM/WqBWe)[10.1093/jnci/djx058](http://dx.doi.org/10.1093/jnci/djx058)[.](http://paperpile.com/b/CxF8UM/WqBWe)

31 [Manichaikul A, Mychaleckyj JC, Rich SS, Daly K, Sale M, Chen W-M. Robust relationship inference in genome-wide association studies. *Bioinformatics* 2010; **26**: 2867–2873.](http://paperpile.com/b/CxF8UM/5aojD)

32 [Danecek P, Auton A, Abecasis G, Albers CA, Banks E, DePristo MA *et al.* The variant call format and VCFtools. *Bioinformatics* 2011; **27**: 2156–2158.](http://paperpile.com/b/CxF8UM/sVz8H)

33 [Kehdy FSG, Gouveia MH, Machado M, Magalhães WCS, Horimoto AR, Horta BL *et al.* Origin and dynamics of admixture in Brazilians and its effect on the pattern of deleterious mutations. *Proc Natl Acad Sci U S A* 2015; **112**: 8696–8701.](http://paperpile.com/b/CxF8UM/9hRNc)

34 [Goldin LR, McMaster ML, Rotunno M, Herman SEM, Jones K, Zhu B *et al.* Whole exome sequencing in families with CLL detects a variant in Integrin β 2 associated with disease susceptibility. *Blood* 2016; **128**: 2261–2263.](http://paperpile.com/b/CxF8UM/VLe3B)

35 [Rotunno M, McMaster ML, Boland J, Bass S, Zhang X, Burdett L *et al.* Whole exome sequencing in families at high risk for Hodgkin lymphoma: identification of a predisposing mutation in the KDR gene. *Haematologica* 2016; **101**: 853–860.](http://paperpile.com/b/CxF8UM/21aa4)

36 [McMaster ML, Sun C, Landi MT, Savage SA, Rotunno M, Yang XR *et al.* Germline mutations in Protection of Telomeres 1 in two families with Hodgkin lymphoma. *Br J Haematol* 2018; **181**: 372–377.](http://paperpile.com/b/CxF8UM/t9x0g)

37 [Auwera GAV der, Van der Auwera GA, Carneiro MO, Hartl C, Poplin R, del Angel G *et al.* From FastQ Data to High-Confidence Variant Calls: The Genome Analysis Toolkit Best Practices Pipeline. Current Protocols in Bioinformatics. 2013; : 11.10.1–11.10.33.](http://paperpile.com/b/CxF8UM/M5HNZ)

38 [Narasimhan V, Danecek P, Scally A, Xue Y, Tyler-Smith C, Durbin R. BCFtools/RoH: a hidden Markov model approach for detecting autozygosity from next-generation sequencing data. *Bioinformatics* 2016; **32**: 1749–1751.](http://paperpile.com/b/CxF8UM/yT7fm)

39 [Kretzmer H, Bernhart SH, Wang W, Haake A, Weniger MA, Bergmann AK *et al.* DNA methylome analysis in Burkitt and follicular lymphomas identifies differentially methylated regions linked to somatic mutation and transcriptional control. *Nat Genet* 2015; **47**: 1316–1325.](http://paperpile.com/b/CxF8UM/pVAvd)

40 [Waszak SM, Tiao G, Zhu B, Rausch T, Muyas F, Rodriguez-Martin B *et al.* Germline determinants of the somatic mutation landscape in 2,642 cancer genomes. bioRxiv. 2017.](http://paperpile.com/b/CxF8UM/gWKxo)<https://edoc.mdc-berlin.de/17576/> [(accessed 14 Jun2019).](http://paperpile.com/b/CxF8UM/gWKxo)

41 [Robinson JT, Thorvaldsdóttir H, Winckler W, Guttman M, Lander ES, Getz G *et al.* Integrative genomics viewer. *Nat Biotechnol* 2011; **29**: 24–26.](http://paperpile.com/b/CxF8UM/iPknT)

42 [Kendig KI, Baheti S, Bockol MA, Drucker TM, Hart SN, Heldenbrand JR *et al.* Sentieon DNASeq Variant Calling Workflow Demonstrates Strong Computational Performance and Accuracy. *Front Genet* 2019; **10**: 736.](http://paperpile.com/b/CxF8UM/hV9w1)

43 [Landrum MJ, Lee JM, Riley GR, Jang W, Rubinstein WS, Church DM *et al.* ClinVar: public archive of relationships among sequence variation and human phenotype. Nucleic Acids Research. 2014; **42**: D980–D985.](http://paperpile.com/b/CxF8UM/Q320M)

44 [Li Q, Wang K. InterVar: Clinical Interpretation of Genetic Variants by the 2015 ACMG-AMP Guidelines. *Am J Hum Genet* 2017; **100**: 267–280.](http://paperpile.com/b/CxF8UM/vQYnl)

45 [Shen R, Seshan VE. FACETS: allele-specific copy number and clonal heterogeneity analysis tool for high-throughput DNA sequencing. Nucleic Acids Research. 2016; **44**: e131–e131.](http://paperpile.com/b/CxF8UM/0tRyF)

46 [Peprah S, Ogwang MD, Kerchan P, Reynolds SJ, Tenge CN, Were PA *et al.* Risk factors for Burkitt lymphoma in East African children and minors: A case-control study in malaria-endemic regions in Uganda, Tanzania and Kenya. *Int J Cancer* 2019. doi:](http://paperpile.com/b/CxF8UM/7SHjv)[10.1002/ijc.32390](http://dx.doi.org/10.1002/ijc.32390)[.](http://paperpile.com/b/CxF8UM/7SHjv)

47 [Maziarz M, Kinyera T, Otim I, Kagwa P, Nabalende H, Legason ID *et al.* Age and geographic patterns of Plasmodium falciparum malaria infection in a representative sample of children living in Burkitt lymphoma-endemic areas of northern Uganda. Malaria Journal. 2017; **16**. doi:](http://paperpile.com/b/CxF8UM/jf1qT)[10.1186/s12936-017-1778-z](http://dx.doi.org/10.1186/s12936-017-1778-z)[.](http://paperpile.com/b/CxF8UM/jf1qT)

48 [Hill AV, Elvin J, Willis AC, Aidoo M, Allsopp CE, Gotch FM *et al.* Molecular analysis of the association of HLA-B53 and resistance to severe malaria. *Nature* 1992; **360**: 434–439.](http://paperpile.com/b/CxF8UM/1TTmN)

49 [Liao H-M, Liu H, Lei H, Li B, Chin P-J, Tsai S *et al.* Frequency of EBV LMP-1 Promoter and Coding Variations in Burkitt Lymphoma Samples in Africa and South America and Peripheral Blood in Uganda. *Cancers*  2018; **10**. doi:](http://paperpile.com/b/CxF8UM/riU1J)[10.3390/cancers10060177](http://dx.doi.org/10.3390/cancers10060177)[.](http://paperpile.com/b/CxF8UM/riU1J)

50 [Rentzsch P, Witten D, Cooper GM, Shendure J, Kircher M. CADD: predicting the deleteriousness of variants throughout the human genome. *Nucleic Acids Res* 2019; **47**: D886–D894.](http://paperpile.com/b/CxF8UM/fjAkA)

51 [Kopanos C, Tsiolkas V, Kouris A, Chapple CE, Albarca Aguilera M, Meyer R *et al.* VarSome: the human genomic variant search engine. *Bioinformatics* 2019; **35**: 1978–1980.](http://paperpile.com/b/CxF8UM/WSnkz)

52 [Richards S, Aziz N, Bale S, Bick D, Das S, Gastier-Foster J *et al.* Standards and guidelines for the interpretation of sequence variants: a joint consensus recommendation of the American College of Medical Genetics and Genomics and the Association for Molecular Pathology. *Genet Med* 2015; **17**: 405–424.](http://paperpile.com/b/CxF8UM/8eDyi)

53 [Karczewski KJ, Francioli LC, Tiao G, Cummings BB, Alföldi J, Wang Q *et al.* The mutational constraint spectrum quantified from variation in 141,456 humans. bioRxiv. 2020; : 531210.](http://paperpile.com/b/CxF8UM/ExVXj)

54 [Kent WJ, Sugnet CW, Furey TS, Roskin KM, Pringle TH, Zahler AM *et al.* The human genome browser at UCSC. *Genome Res* 2002; **12**: 996–1006.](http://paperpile.com/b/CxF8UM/y5FJe)

55 [Mozzi A, Forni D, Cagliani R, Pozzoli U, Clerici M, Sironi M. Distinct selective forces and Neanderthal introgression shaped genetic diversity at genes involved in neurodevelopmental disorders. *Sci Rep* 2017; **7**: 6116.](http://paperpile.com/b/CxF8UM/69CZT)

56 [Holowaty MN, Frappier L. HAUSP/USP7 as an Epstein-Barr virus target. *Biochem Soc Trans* 2004; **32**: 731–732.](http://paperpile.com/b/CxF8UM/RAN6r)

57 [Nishiyama M, Skoultchi AI, Nakayama KI. Histone H1 recruitment by CHD8 is essential for suppression of the Wnt-β-catenin signaling pathway. *Mol Cell Biol* 2012; **32**: 501–512.](http://paperpile.com/b/CxF8UM/loot7)

58 [Judson SC, Henle W, Henle G. A cluster of Epstein-Barr-virus-associated American Burkitt’s lymphoma. *N Engl J Med* 1977; **297**: 464–468.](http://paperpile.com/b/CxF8UM/cjVU1)

59 [Johnston WT, Mutalima N, Sun D, Emmanuel B, Bhatia K, Aka P *et al.* Relationship between Plasmodium falciparum malaria prevalence, genetic diversity and endemic Burkitt lymphoma in Malawi. *Sci Rep* 2014; **4**: 3741.](http://paperpile.com/b/CxF8UM/e6Z7a)

# Section 7. Supplementary figures


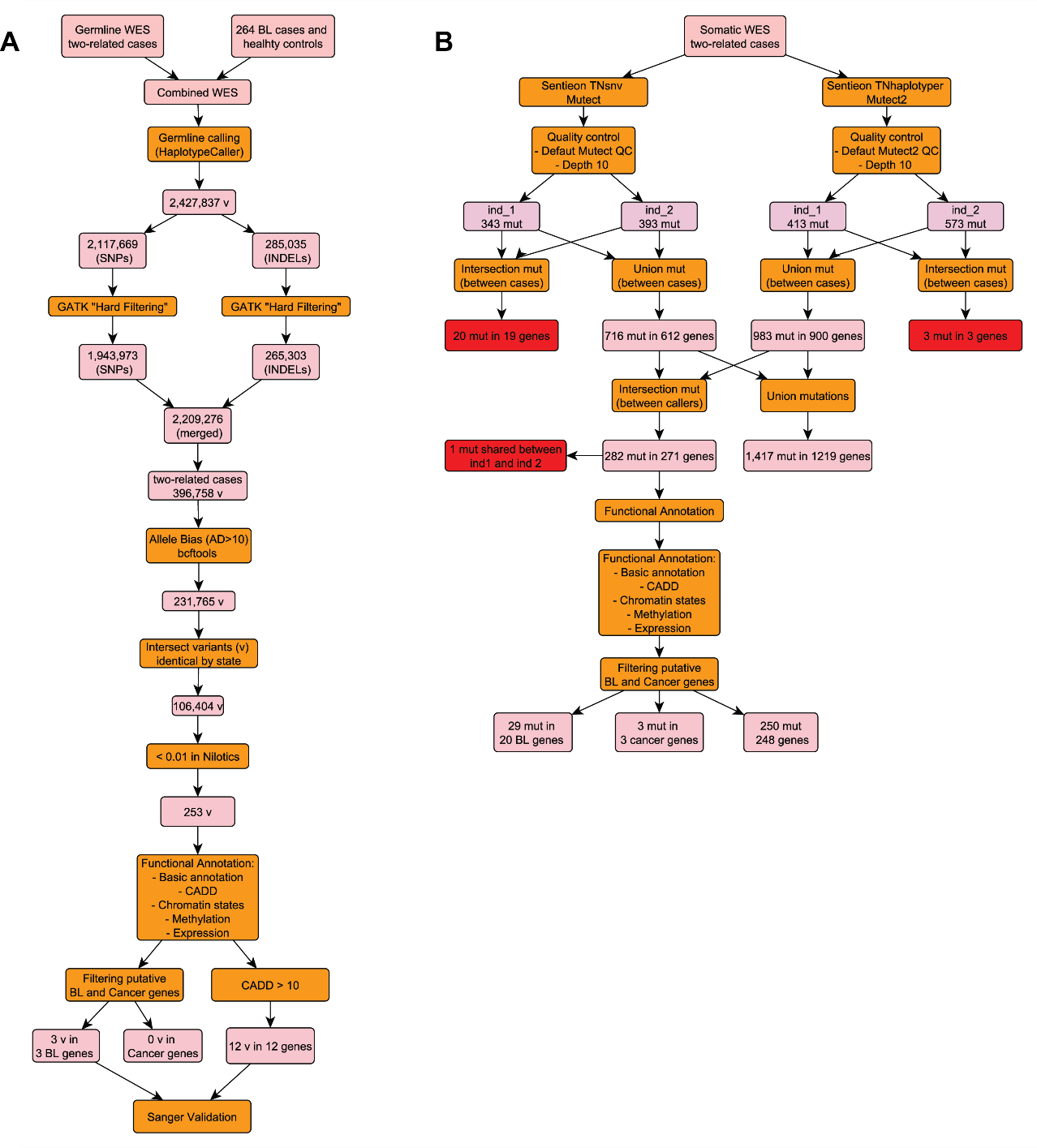


**Fig. S1. Whole-Exome Sequencing (WES) analysis flowchar**t. Analysis of the (A) germline DNA and (B) somatic WES data. v = variants, mut = mutations, GATK = **G**enome**A**nalysis**T**ool**K**it and CADD = Combined Annotation Dependent Depletion.


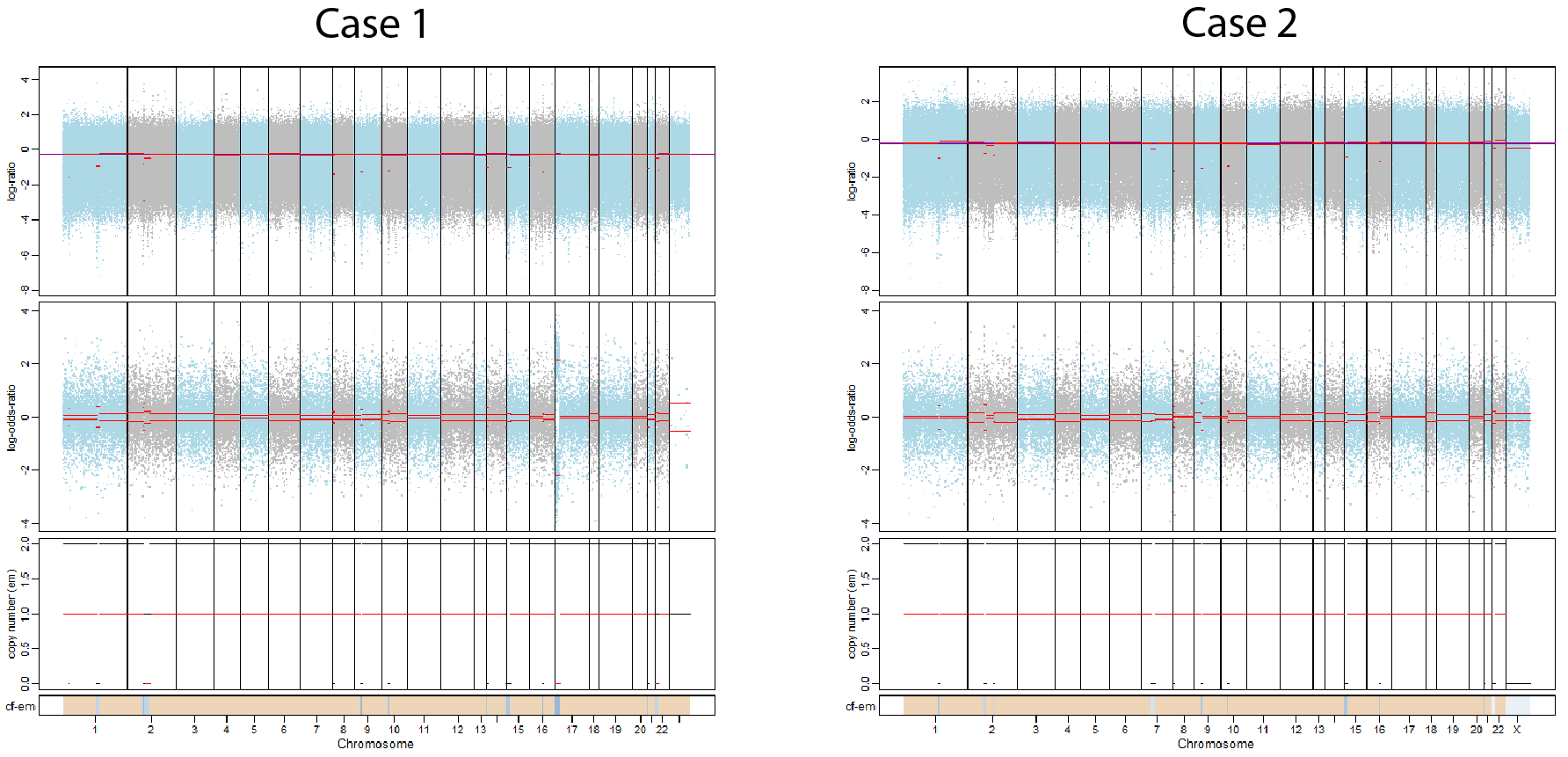


**Fig. S2. Allele-Specific Copy Number Estimates from Tumor Sequencing (FACETS) analysis of tumor-germline DNA whole-exome sequencing data from the second-degree relatives with** **eBL.** The top panel displays total copy number log-ratio (logR) and the second panel displays allele-specific log-odds-ratio data (logOR) with chromosomes alternating in blue and gray. The third panel plots the corresponding integer (total, minor) copy number calls. The estimated cellular fraction (cf) profile is plotted at the bottom, revealing both clonal and sub-clonal copy number events.

**
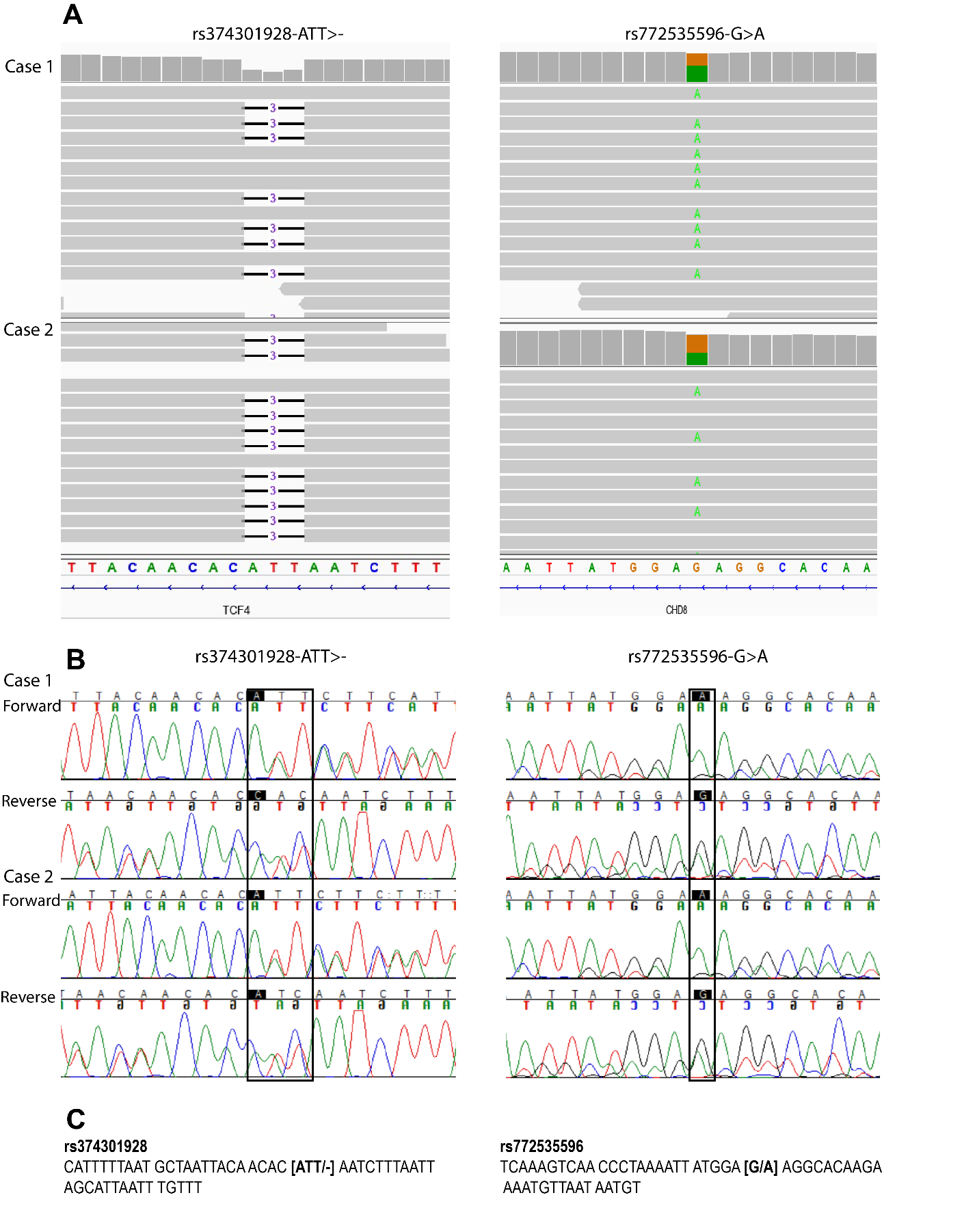
**

**Fig. S3. The variants rs374301928 in *TCF4* and rs772535596 in *CHD8* in the germline DNA of the second-degree relatives with eBL.** A) WES discovery analysis visualized by the Integrative Genomics Viewer (IGV) showing individual sequencing reads with variable sites indicated by the number (nucleotides inserted or deleted) or by the letters. B) Validation analysis represented by Sanger sequencing chromatograms visualized using Sequencher 5.2.4. C) Genomic context of rs374301928 and rs772535596 variants. Both variants were scored as heterozygous in the germline DNA of eBL case 1 and case 2, by WES and Sanger sequencing.


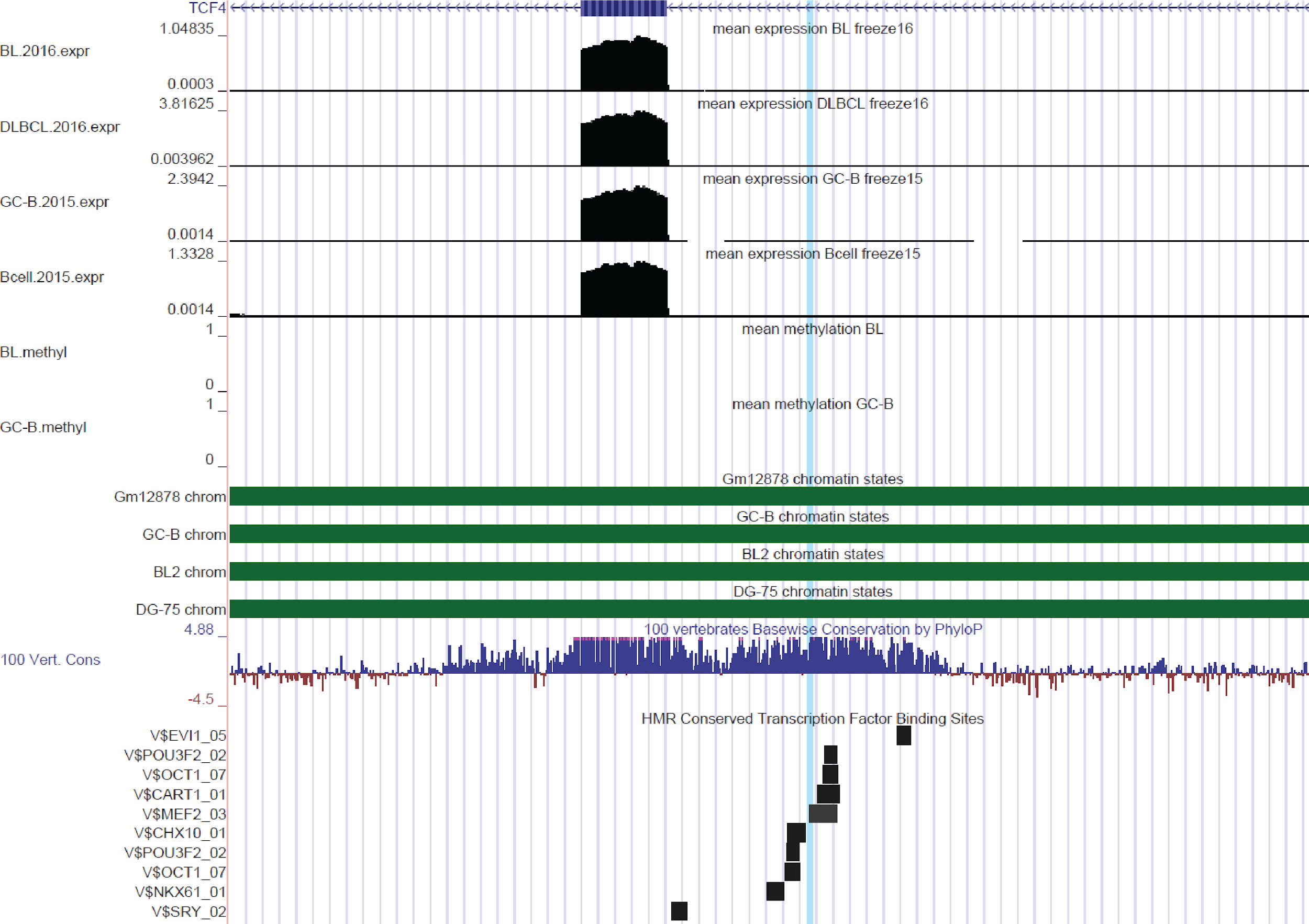


**Fig S4. Genomic landscape around the *TCF4* intronic deletion rs374301928.** UCSC genome browser display (GRCh37/hg19) of the region chr18:52,928,502-52,929,256; accessed 04/26/2020 at <http://genome-euro.ucsc.edu>). The location of the rs374301928 deletion within the 754 bp region is highlighted in light blue. Under the reference sequence track for *TCF4* also shown: gene expression in sBL and GC-B-cells (Kretzmer et al. 2015 [Ref #39]); Lopez et al. 2019 [Ref #12]), intra-species DNA conservation, conserved transcription factor binding sites and chromatin states in normal B-cells and BL cell lines (green = transcription; Kretzmer et al. 2015 [Ref #39]). The deletion rs374301928 is embedded in a highly conserved genomic region with various binding sites for transcription factors active in B-cells.
